# Proteomic Identification of S-Nitrosylated Golgi Proteins: New Insights into Endothelial Cell Regulation by eNOS-Derived NO

**DOI:** 10.1371/journal.pone.0031564

**Published:** 2012-02-21

**Authors:** Panjamaporn Sangwung, Todd M. Greco, Yanzhuang Wang, Harry Ischiropoulos, William C. Sessa, Yasuko Iwakiri

**Affiliations:** 1 Section of Digestive Diseases, Department of Internal Medicine, Yale University School of Medicine, New Haven, Connecticut, United States of America; 2 Department of Pharmacology and Children's Hospital of Philadelphia Research Institute, Children's Hospital of Philadelphia and University of Pennsylvania, Philadelphia, Pennsylvania, United States of America; 3 Department of Molecular Biology, Princeton University, Princeton, New Jersey, United States of America; 4 Department of Molecular, Cellular and Developmental Biology, University of Michigan, Ann Arbor, Michigan, United States of America; 5 Department of Pharmacology, Yale University School of Medicine, New Haven, Connecticut, United States of America; Istituto Dermopatico dell'Immacolata, Italy

## Abstract

**Background:**

Endothelial nitric oxide synthase (eNOS) is primarily localized on the Golgi apparatus and plasma membrane caveolae in endothelial cells. Previously, we demonstrated that protein S-nitrosylation occurs preferentially where eNOS is localized. Thus, in endothelial cells, Golgi proteins are likely to be targets for S-nitrosylation. The aim of this study was to identify S-nitrosylated Golgi proteins and attribute their S-nitrosylation to eNOS-derived nitric oxide in endothelial cells.

**Methods:**

Golgi membranes were isolated from rat livers. S-nitrosylated Golgi proteins were determined by a modified biotin-switch assay coupled with mass spectrometry that allows the identification of the S-nitrosylated cysteine residue. The biotin switch assay followed by Western blot or immunoprecipitation using an S-nitrosocysteine antibody was also employed to validate S-nitrosylated proteins in endothelial cell lysates.

**Results:**

Seventy-eight potential S-nitrosylated proteins and their target cysteine residues for S-nitrosylation were identified; 9 of them were Golgi-resident or Golgi/endoplasmic reticulum (ER)-associated proteins. Among these 9 proteins, S-nitrosylation of EMMPRIN and Golgi phosphoprotein 3 (GOLPH3) was verified in endothelial cells. Furthermore, S-nitrosylation of these proteins was found at the basal levels and increased in response to eNOS stimulation by the calcium ionophore A23187. Immunofluorescence microscopy and immunoprecipitation showed that EMMPRIN and GOLPH3 are co-localized with eNOS at the Golgi apparatus in endothelial cells. S-nitrosylation of EMMPRIN was notably increased in the aorta of cirrhotic rats.

**Conclusion:**

Our data suggest that the selective S-nitrosylation of EMMPRIN and GOLPH3 at the Golgi apparatus in endothelial cells results from the physical proximity to eNOS-derived nitric oxide.

## Introduction

Nitric oxide (NO) conveys specific cellular signals via S-nitrosylation despite its highly reactive and diffusible nature [1], [2]. S-nitrosylation is a post-translational modification of cysteine-thiols to form nitroso-thiols [3]. One of the most important factors that specify the targets of S-nitrosylation is the compartmentalization of NO synthase (NOS, a source of NO^•^) with its target proteins for S-nitrosylation [1]. This compartmentalization allows for the generation of relatively high local NO concentrations in the vicinity of the target proteins, which enables the formation of S-nitrosocysteine. Endothelial NOS (eNOS) is unique among the NOS family members as it is localized mainly to specific intracellular membrane domains in endothelial cells, including the cytoplasmic side of the Golgi apparatus and the plasma membrane caveolae [4]–[6].

Previously we showed that the localization of eNOS in the cell is an important determinant of protein S-nitrosylation [7]. Using a mutant eNOS that was targeted to the nucleus and wild-type eNOS that is localized on the Golgi apparatus, we demonstrated that protein S-nitrosylation occurs where eNOS is localized. Further, we presented that localization of eNOS at the Golgi apparatus influences Golgi functions such as protein trafficking in endothelial cells. Specifically, eNOS localized at the Golgi reduces the speed of protein transport from the endoplasmic reticulum (ER) to the Golgi apparatus and from the Golgi to the plasma membrane [7].

The Golgi is a membrane organelle that plays essential roles in post-translational modifications such as glycosylation. It is a dynamic structure and its membranes are constantly remodeled during cell growth, migration and division. Therefore, eNOS localization to the Golgi apparatus implies a unique property of this enzyme in regulating Golgi function, likely through S-nitrosylation of Golgi membrane proteins [8]. Thus, identification of S-nitrosylated proteins on the Golgi membranes is required for understanding endothelial cell function regulated by eNOS-derived NO.

In this study, we performed a proteomic analysis [9] of Golgi membranes isolated from rat livers and identified 78 putative S-nitrosylated proteins and the target cysteine residues. Nine of them were Golgi resident and Golgi/endoplasmic reticulum (ER)-associated proteins that are largely representative of critical Golgi-related cellular functions such as protein trafficking and glycosylation. Among them, we confirmed that at least two proteins, extracellular matrix metalloproteinase inducer (EMMPRIN) and Golgi phosphoprotein 3 (GOLPH3), are endogenously S-nitrosylated and co-localized with eNOS at the Golgi apparatus in endothelial cells, providing strong evidence for compartmentalization-induced selective S-nitrosylation.

## Materials and Methods

### Plasmids

A yellow fluorescent protein (YFP)-tagged rat EMMPRIN cDNA construct was a kind gift from Drs. Andrew P. Halestrap and Marieangela C. Wilson (University of Bristol, UK) [10]. A red fluorescent protein (RFP)-tagged wild-type eNOS (WTeNOS-RFP) was generated as described [7]. An HA-tagged human GOLPH3 in pBABE-Puro vector was obtained from Addgene (Cambridge, MA).

### Isolation of an enriched Golgi membrane fraction

**Figure 1:**
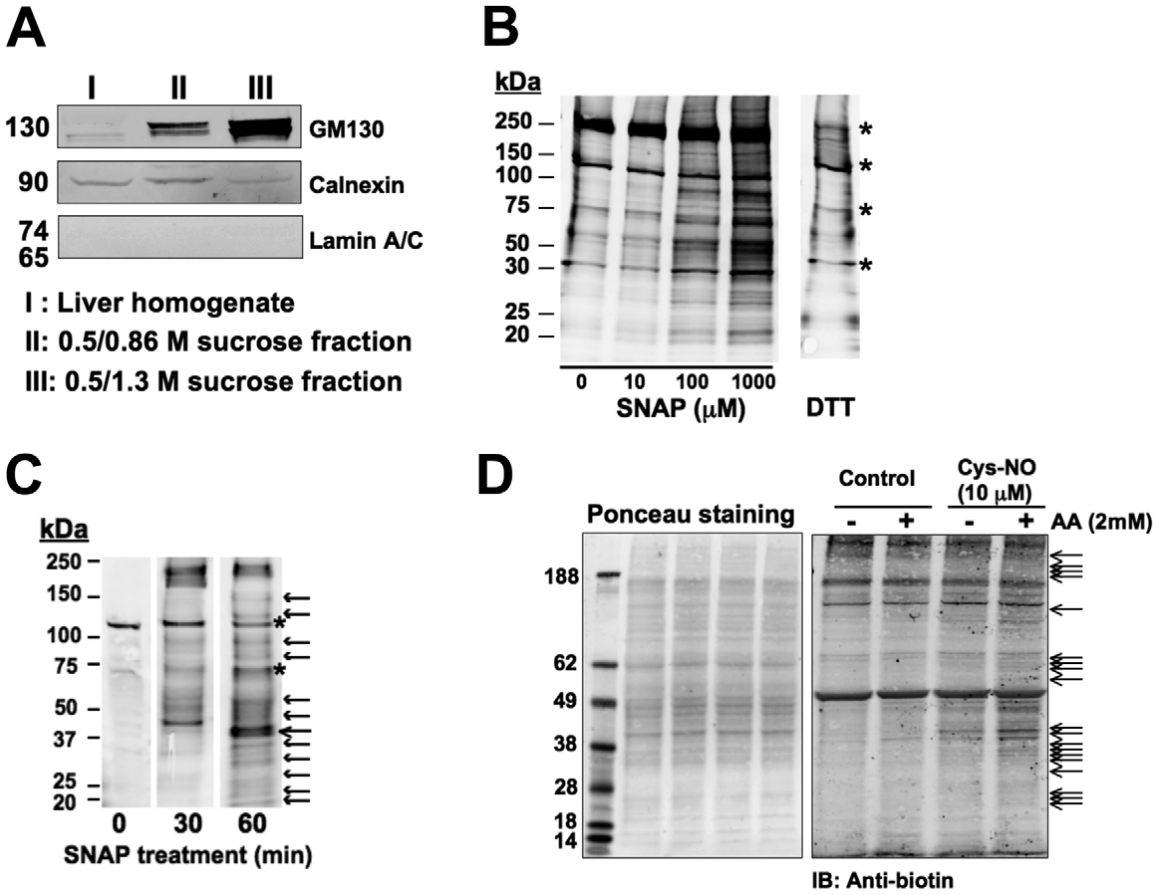
Proteins at the Golgi apparatus can be targets for S-nitrosylation. (**A**) Golgi membrane enrichment and its purity were assessed by Western blot with a Golgi marker (GM130), an endoplasmic reticulum (ER) marker (Calnexin) and a nuclear marker (Lamin A/C). Fraction III of the preparation showed a successful enrichment and purity of Golgi membranes and was used in this study. (**B**) S-nitrosylated Golgi membrane proteins were increased dose-dependently in response to the addition of an NO donor, S-nitroso-N-acetylpenicillamine (SNAP). Golgi membranes were treated with indicated concentrations of SNAP *in vitro* at room temperature (RM) for 30 min. Protein S-nitrosylation was assessed by the biotin-switch assay. Asterisks indicate endogenous biotin-containing proteins, thus considered as non-specific bands. Dithiothreitol (DTT), which cleaves nitroso-cysteine bonds, serves as a negative control. Shown are representative blots from 3 independent experiments. (**C**) S-nitrosylated Golgi membrane proteins were increased time-dependently in response to 100 µM SNAP *in vitro* for indicated incubation times (0, 30 and 60 min). Arrows indicate those proteins increased in response to SNAP. Asterisks indicate endogenous biotin-containing proteins, thus considered as non-specific bands. Shown are representative images from 3 independent experiments. (**D**) The sample quality was verified before performing a proteomic analysis. Successful biotinylation (i.e., S-nitrosylation) by the biotin-switch assay was determined by Western blot. Golgi membranes were incubated with or without 10 µM S-nitrosocysteine (Cys-NO) for 15 min at 37°C. Then, the biotin-switch assay was performed in the presence or absence of 2 mM ascorbic acid (AA) (right panel). Proteins were separated by SDS-PAGE and an equal protein loading to each lane was confirmed by Ponceau staining (left panel). Subsequent Western blotting using an anti-biotin antibody detected biotinylated proteins (right panel). Arrows indicate unique bands of biotinylated proteins that appeared in the presence of AA. Those biotinylated proteins increased by Cys-NO treatment (the 4^th^ lane in the right panel) were identified by mass spectrometry.

Rat liver Golgi membranes were isolated from adult male Sprague-Dawley rats as described [11]. Briefly, male rat livers were homogenized using a 150-µm mesh sieve in a buffer containing 100 mM potassium phosphate buffer, pH6.7, 5 mM magnesium chloride, 0.5 M sucrose and protease inhibitors. The homogenates were layered over 0.86 M sucrose buffer, followed by a layer of 0.25 M sucrose buffer, and then centrifuged at 100,000×*g* for 1 hour at 4°C. After the centrifugation, the Golgi membrane fraction was accumulated at the interface between 0.5 M and 0.86 M sucrose buffers (Fraction II in Figure 1A). This Golgi membrane fraction was then adjusted to 0.25 M sucrose, placed on 0.5 M (middle layer) and 1.3 M sucrose (bottom layer) buffers, and then centrifuged at 8,000×*g* for 30 min at 4°C. The Golgi membrane was collected at the 0.5 M/1.3 M sucrose interface (Fraction III in Figure 1A).

### Quantification of protein S-nitrosylation

S-nitrosocysteine (Cys-NO) formation was determined by tri-iodide reduction coupled to chemiluminescence detection using a Sievers 280 NO analyzer as described [9]. Approximately a total of 400 µg Golgi membrane proteins was used. Golgi membrane proteins were incubated with different concentrations of NO donors, Cys-NO and diethylamine NONOate (DEANO), for 10 min at 37°C, and followed by acetone precipitation for 1 hour. Protein pellets were resuspended in an isolation buffer and adjusted to a concentration of 1 mg/ml. The isolation buffer contained 20 mM HEPES, pH7.7, 1 mM diethylene triamine pentaacetic acid (DTPA), 0.1 mM neocuproine, 1% Triton X-100 and 2 mM methyl methanethiosulfonate (MMTS). Protein samples were then split into two equal volumes; one sample was added 1/10 volume of 1% sulfanilamide (SNA)/glacial acid and incubated on ice for at least 15 min to remove nitrite contamination. The other sample was added 1/10 volume of 1% SNA/glacial acid and 30 mM HgCl_2_, incubated at room temperature (RT) for 10 min, and then placed on ice. S-nitrosoglutathione (GSNO) was used as a standard. Approximately 15 to 50 µg of Golgi membrane proteins were injected into a reaction vessel containing 5 ml of 60 mM potassium iodide and 10 mM iodine in glacial acetic acid at 37°C.

### Biotin-switch assay

Approximately a total of 2.0 mg Golgi membrane proteins was used for the biotin-switch assay [12] necessary to perform a proteomic analysis described below, while an approximately 200 µg of Golgi membrane proteins was used per treatment group for dose-response and time-course experiments with an NO donor, S-nitroso-N-acetyl-D,L-penicillamine (SNAP). Golgi membrane proteins were also treated with or without another NO donor, nitrosocysteine (Cys-NO, 10 µM) for 15 min at 37°C. After cold acetone precipitation, pellets were resuspended in a lysis buffer (HEN buffer containing 1%Triton X-100 and protease inhibitors) to obtain 1 mg/ml. Samples were adjusted to 0.5 mg/ml containing 2.5% SDS and 200 mM MMTS and incubated at 50°C for 20 min with every 5 min of vortexing to block free thiols. After blocking, cell extracts were precipitated with two volumes of −20°C acetone, incubated at −20°C for 20 min, centrifuged at 12,000×*g* for 10 min at 4°C, washed four times with acetone, and resuspended in 0.2 ml of HENS buffer (25 mM HEPES, pH7.7/0.1 mM EDTA/0.01 mM neocuproine/1% SDS). Then, 0.4 mM biotin-HPDP and 5 mM ascorbate were added and incubated at 25°C for 1 hour while rotating. As a control for non-specific HPDP labeling, ascorbate was omitted. Proteins were precipitated with acetone. Samples in which protein digestion was performed for further proteomic analysis were resuspended in 0.45 ml of 0.1 M ammonium bicarbonate and 0.5% SDS [9]. Otherwise, samples were processed for detection of biotinylated proteins as previously described [12], [13].

### Protein digestion and affinity peptide capture

Biotinylated protein samples were incubated with trypsin (1∶30 enzyme/protein ratio) at 37°C for 18–24 hours in the dark as described [9]. The samples were passed through Ultra-free-MC 10-kDa cutoff filters that had previously been rinsed with methanol and washed with H_2_O. The filtrate containing the peptides was recovered and incubated with approximately 50 µl of dry, washed streptavidin-agarose beads per mg of initial protein for 30 min with gentle mixing. The samples were centrifuged at 5,000×*g* for 5 min, and the supernatants were discarded. The beads were washed five times with 10 volumes of 1 M ammonium bicarbonate, followed by five washes with 10 volumes of deionized water. Between washes, the samples were centrifuged at 1,000×*g* for 1 min. An elution buffer containing 70% formic acid was incubated with the beads for 30 min with gentle mixing. The captured peptides were recovered by centrifuging the beads at 5,000×*g* for 4 min and collecting the supernatants. The captured peptides were evaporated to approximately 5 µl *in vacuum*, resuspended in 20 µl of 0.1% formic acid, and desalted using Zip-Tips.

### Analysis by LC-MS/MS

Desalted samples were analyzed on a Thermo LTQ linear trap mass spectrometer equipped with a Thermo micro electrospray source, a Thermo Surveyor pump and autosampler (Thermofisher Scientific, San Jose, CA) as described [9]. MS/MS spectra were searched with SEQUEST (Bioworks Browser 3.1 SR1) against the rat NCBI RefSeq database concatenated with corresponding reverse sequences. Cysteine modification by MMTS (+46 atomic mass units) and by biotin-HPDP (+428 atomic mass units) was specified as variable modifications. Peptide spectrum matches (PSMs) were empirically filtered by SEQUEST cross correlation scores (X_c_) using PSMs assigned to the reverse database to achieve an estimated false discovery rate of <5%. RefSeq accessions were mapped on to UniProt UniRef accessions.

### Immunofluorescence

Transfected COS-7 cells were fixed with 4% paraformaldehyde in PBS (pH7.4) for 10 min at room temperature (RT) or with cold-methanol for 10 min at 4°C. After wash, the cells were permeabilized for 10 min at RT with PBS containing 0.1% Triton-X 100. The cells were blocked for 30 min at RT with PBS containing 5% Donkey serum and 1% Bovine serum albumin (BSA). Afterward, the cells were incubated for 2 hours at RT with rat anti-HA (1∶500; Roche Applied Science, Indianapolis, IN, Cat#:11867423001) for HA-GOLPH and rabbit anti-eNOS antibodies (1∶10; Novus Biologicals, Littleton, CO, Cat#:NB120-15280). After wash, the cells were incubated for 1 hour at RT with secondary antibodies, Alexa Flour 568 anti-rat IgG and Alexa Flour 647 anti-rabbit IgG (1∶500; Invitrogen, Grand Island, NY).

Immuno-labeling of eNOS (rabbit, 1∶10; Novus Biologicals, Littleton, CO, Cat#:NB120-15280), EMMPRIN (goat, 1∶100; Santa Cruz, CA, Cat#:sc-9757), GM130 (mouse, 1∶500; BD Biosciences, CA, Cat#:610822) and GOLPH 3 (rabbit, 1∶100; Abcam, MA, Cat#:AB91492) was performed in bovine aortic endothelial cells (BAECs) grown in 8-well slides. The cells were fixed with 4% paraformaldehyde for 10 min at 4°C, permeabilized with 0.1% Triton X-100/PBS for 15 min at RT, blocked with 5% donkey serum/0.3% BSA/PBS for 30 min at RT, and incubated with primary antibodies overnight at 4°C. Then, the cells were incubated with secondary antibodies conjugated to 488 or 568 (1∶500 in PBS) for 1 hour at RT. After wash, the cells were mounted with DAPI media (Invitrogen, CA) for nuclear staining according to the manufacturer's instruction. Images were taken using a Nikon E800 Microscope with a Plan-Fluorchromat 40×/0.75 objective (Nikon, Melville, NY).

### Immunoprecipitation

Bovine aortic endothelial cells (BAECs) were grown in 15 cm plates. After overnight serum withdrawal (typically ∼16 hours), the cells were stimulated with 10 µM calcium ionophore (A23187) for 30 min at 37°C. The cells (∼80% confluent) were collected in 1 ml lysis buffer containing 10% glycerol, 50 mM Tris-HCl, 0.1 mM EGTA, 0.1 mM EDTA, 5 mM sodium fluoride, 1 mM sodium pyrophosphate, 1 mM sodium vanadate, 1 mM 4-(2-aminoethyl)-benzenesulfonyl fluoride, a protease inhibitor cocktail tablet (Roche Diagnostics, Mannheim, Germany), 1% (vol/vol) Nonidet P-40, 0.1% SDS, and 0.1% deoxycholate; pH7.5. Lysates were incubated for 1 hour at 4°C on a rotating mixer, and centrifuged at 13,000 rpm for 10 min at 4°C. Approximately 20 µl of Protein G slurry was added to each sample, adjusted to 1 mg protein/500 µl lysis buffer, incubated for 1 hour at 4°C, and then centrifuged at 11,400 rpm for 20 sec at 4°C to remove non-specific binding to beads. The supernatants were collected to new tubes, added 5 µl (1∶100) of S-nitrosocysteine antibody (rabbit, Sigma, St. Louis, MO, Cat#:N5411) [14] or 2 µg of EMMPRIN antibody (goat, Santa Cruz Biotechnology, Santa Cruz, CA, Cat#:sc-9757), and then incubated overnight at 4°C on a rotating mixer. For a negative control, 4 µg of rabbit or goat IgG was used. Then, ∼50 µl of Protein G slurry was added, incubated for 1 hour at 4°C, and centrifuged at 11,400 rpm for 20 sec at 4°C. Beads were washed 5 times with 1 ml of washing buffer (0.1 mM EDTA, 0.1 mM EGTA, 50 mM Tris-HCl, pH 7.5). In an additional set of experiments we included 150 mM NaCl into the wash buffer. Proteins bound to the antibody were eluted with 50 µl of 2×SDS sample buffer containing 2-mercaptoethanol, boiled at 95°C for 10 min, and stored at −80°C until analyzed by Western blot. Antibodies used for Western blot analysis included rabbit anti-S-nitrosocysteine (1∶1000, Sigma, St. Louis, MO, Cat#:N5411), goat anti-EMMPRIN (1∶500, Santa Cruz Biotechnology, Cat#:sc-9757), mouse anti- eNOS (1∶1000, BD Biosciences, San Jose, CA, Cat#:610296), and Golgi phosphoprotein 3 (GOLPH3, rabbit, 1∶500, Abcam, Cambridge, MA, Cat#:AB91492). Fluorophore-conjugated secondary antibodies (either 680 nm or 800 nm emission) were incubated with membranes for 1 hour at RT. Detection and quantification of bands were performed using the Odyssey Infrared Imaging System (Li-Cor Biotechnology, Lincoln, NE).

### Generation of cirrhotic rats and isolation of the aorta from cirrhotic rats

Male Sprague-Dawley rats (Harlan Sprague-Dawley Laboratories, Indianapolis, IN), weighing 100–125 g, were exposed to carbon tetrachloride (CCl_4_) by inhalation for 12 weeks as described [15]. The aorta from three cirrhotic and three age-matched control rats were used for analysis. Vessels were lysed in the lysis buffer mentioned above and the biotin switch assay was performed using an equal amount of proteins (500 µg) per group. Biotinylated proteins (i.e., S-nitrosylated proteins) were purified using streptavidin agarose beads and detected by Western blot. All procedures were performed in accordance with the “Principles of Laboratory Animal Care” and were approved by the Animal Care and Use Committee at the Veterans Affairs Healthcare System of Connecticut (Protocol#: YI0002).

## Results

### Golgi membrane proteins were successfully enriched

We isolated Golgi membranes from rat livers. We chose this tissue for several reasons. First, rat liver is the most commonly used organ for studies of Golgi function and the protocol for isolation of Golgi membranes is well established [11]. Second, in order to perform the biotin-switch assay and the subsequent proteomic analysis, a large quantity of Golgi membrane proteins (at least 1.0–2.0 mg per treatment) was required.

The yield of Golgi membrane proteins was approximately 1.0–2.0 mg per liver. An examination of Golgi membrane proteins by Western blotting showed a successful enrichment of the final Golgi membrane fraction, which was used for further analysis of S-nitrosylated Golgi membrane proteins (Figure 1A). There was slight contamination of endoplasmic reticulum (ER), while contamination of nuclear membranes was very minimal (Figure 1A).

### Golgi membrane proteins are S-nitrosylated in response to NO donors

To investigate S-nitrosylation of Golgi membrane proteins, we first determined S-nitrosylated protein levels by the biotin-switch assay (Figures 1B and C) and the tri-iodide chemiluminescent method (Figure S1). A dose and time-dependent increase in S-nitrosylated proteins in response to SNAP treatment of Golgi membranes was determined (Figures 1B and C). Similarly, S-nitrosylation of Golgi membrane proteins was increased in response to treatment with S-nitrosocysteine (Cys-NO) and DEANO (Figure S1). As reported previously [9], Cys-NO was a more effective S-nitrosylating agent than DEANO.

### Proteomic analysis and identification of S-nitrosylated Golgi and Golgi/ER-associated proteins

Based on the results in Figure S1 and our previous experience in exposing cells to different concentrations of Cys-NO [9], we decided to carry out S-nitrosylation of Golgi membrane proteins for a proteomic analysis using 10 µM Cys-NO, a concentration that results in the formation of S-nitrosylated proteins without compromising cellular functionality [8]. Before conducting a proteomic analysis, we ran a Western blot after the biotin switch assay for Golgi membrane proteins to verify whether S-nitrosylated proteins were biotinylated in the presence or absence of ascorbic acid (AA) during the biotin-switch assay. In the presence of AA, nitroso-thiol residues (i.e., S-nitrosylated cysteine residues) are converted to thiol residues, which then react with biotin-HPDP, a thiol-specific biotinylating reagent. Therefore, biotinylation that occurs in the absence of AA can be considered non-specific. As indicated by arrows in Figure 1D (the 4th lane on the right membrane), the Cys-NO treatment in the presence of AA resulted in unique bands of biotinylated (i.e., previously S-nitrosylated) proteins. Biotinylated proteins were subjected to tryptic digestion followed by streptavidin-based enrichment of biotinylated peptides, and subsequent tandem mass spectrometry analysis. Biotinylated peptides that were identified from samples treated with biotin-HPDP alone (in the absence of AA) were considered non-specific and were excluded.

We identified 78 putative S-nitrosylated proteins from our Golgi membrane samples (Table S1), nine of which were Golgi resident and Golgi/ER-associated proteins (Table 1), while the rests were considered proteins in transit through the Golgi apparatus. This proteomic analysis also allowed us to identify the site where S-nitrosylation occurs on the protein.

**Table 1:** Putative S-nitrosylated Golgi membrane proteins in rats.

	Function/protein name	Uniprot Accession	Sequence	Residue
**Extracellular Matrix Remodeling**				
1	EMMPRIN/CD147/Basigin	P26453	R.SGEYSC#IFLPEPVGR.G	87
**Signal Transduction/Golgi Structure**				
2	Golgi phosphoprotein 3	Q9ERE4	R.QLLDLDPEVEC#LK.A	288
**Protein Trafficking**				
3	ERGIC-53 (p58)	Q62902	K.NNPAIVVVGNNGQINYDHQNDGATQALASC#QR.D	190
4	Transmembrane emp24 domain-containing protein 9 (TMED9)	Q5I0E7	K.C#FIEEIPDETMVIGNYR.T	28
**Glycosylation**				
5	Alpha-6-fucosyltransferase	Q6EV76	R.YATGGWETVFRPVSETC#TDR.S	266
6	Fukutin related protein	Q4KLJ4	R.C#DALDGDAVLLMR.S	191
7	Polypeptide N-acetylgalactosaminyltransferase11	Q6P6V1	K.GYVGMAIC#DGSSSQQWR.L	596
8	RCG57892/D-glucuronic acid C5-epimerase	D3ZIK0	K.YEEIDC#LINDEHTIR.G	109
**Golgi Structure**				
9	Giantin/MacroGolgin/Rat GCP360(rat)	Q63714	R.LKQVQVEIC*ELK.K K.C*REHENNLEGIIK.Q	1399 2267

### EMMPRIN and GOLPH3 are S-nitrosylated in endothelial cells

**Figure 2:**
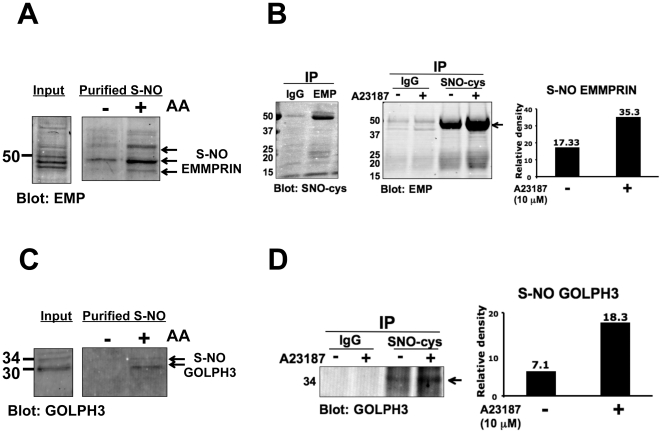
EMMPRIN and GOLPH3 are S-nitrosylated in endothelial cells. (**A**) S-nitrosylation of EMMPRIN was detected by the biotin-switch assay in bovine aortic endothelial cells (BAECs). Input refers to EMMPRIN levels in lysates before performing the biotin-switch assay. Specificity of biotinylation was confirmed by incubating samples in the presence or absence of 2 mM ascorbic acid (AA) during the biotin-switch assay. Arrows indicate S-nitrosylated EMMPRIN (EMP). Shown are representative results from 3 independent experiments. (**B**) S-nitrosylated EMMPRIN levels in BAECs in the basal (non-stimulated, left panel) and after eNOS stimulation with 10 µM of a calcium ionophore (A23187) for 30 min to promote NO production (middle panel). Arrow indicates S-nitrosylated EMMPRIN. BAEC lysates immunoprecipitated (IP) with EMMPRIN (EMP, left panel) or S-nitroso-cysteine (SNO-cys, middle panel) antibodies were blotted with SNO-cys or EMMPRIN antibodies, respectively. The bar graph on the right panel shows band intensities of S-nitrosylated EMMPRIN (arrow in the middle panel) in BAECs stimulated with or without A23187. The blots are representative images from 3 independent experiments. (**C**) S-nitrosylation of GOLPH3 was detected by the biotin-switch assay in BAECs. Input refers to GOLPH3 levels in lysates before performing the biotin-switch assay. Specificity of biotinylation was confirmed by incubating samples in the presence or absence of 2 mM ascorbic acid (AA) during the biotin-switch assay. Arrows indicate S-nitrosylated GOLPH3. The blots are representative images from 3 independent experiments. (**D**) S-nitrosylated GOLPH3 levels in BAECs after eNOS stimulation with 10 µM of A23187 for 30 min to promote NO production (left panel). BAEC lysates immunoprecipitated with S-nitrosocysteine (SNO-cys) antibodies were blotted for GOLPH3. The graph on the right panel shows band intensities of S-nitrosylated GOLPH3 (arrow in the left panel) in BAECs stimulated with or without A23187. Shown are representative results from 3 independent experiments.

Based on availability and specificity of antibodies, we chose two proteins from Table 1, EMMPRIN (also known as CD147 or basigin), an inducer of matrix metalloproteinases (MMPs) that are involved in vascular remodeling [16]–[18], and Golgi phosphoprotein 3 (GOLPH3), a Golgi-localized oncoprotein implicated in protein trafficking, receptor recycling, and glycosylation [19], and confirmed that these proteins are S-nitrosylated in endothelial cells. We performed the biotin-switch assay coupled with Western blot for lysates collected from bovine aortic endothelial cells (BAECs) that were treated with a calcium ionophore, A23187, a well-known agonist of eNOS activation. Figure 2A shows that EMMPRIN is S-nitrosylated. We then tested whether EMMPRIN is endogenously S-nitrosylated in unstimulated BAECs. For this experiment, we immunoprecipitated endothelial cell lysates with an S-nitrosocysteine antibody or nonspecific rabbit IgG followed by Western blotting for EMMPRIN. As shown in Figure 2B (left panel), EMMPRIN was S-nitrosylated at a detectable level without eNOS stimulation (i.e., at the basal level) and was increased in response to eNOS stimulation (Figure 2B, middle and right panels, also see Figure S3).

S-nitrosylation of GOLPH3 was also confirmed in endothelial cell lysates by the biotin-switch assay (Figure 2C). Like EMMPRIN, GOLPH3 was S-nitrosylated at the basal level (without A23187) and S-nitrosylation of GOLPH3 increased about 3-fold in response to A23187 treatment in endothelial cells (Figure 2D). Collectively, these observations suggest that EMMPRIN and GOLPH3 are S-nitrosylated in endothelial cells at their basal levels and that S-nitrosylation of these proteins increases upon eNOS stimulation.

### EMMPRIN and GOLPH3 are co-localized with eNOS at the Golgi apparatus in endothelial cells

**Figure 3:**
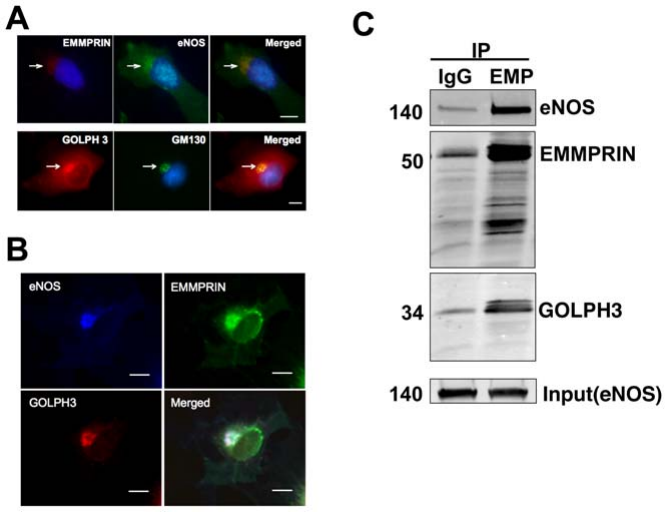
EMMPRIN, GOLPH3 and eNOS are co-localized at the Golgi apparatus in endothelial cells. (**A**) Immunolabeling of EMMPRIN, eNOS, GOLPH3 and GM130 (a Golgi marker) in bovine aortic endothelial cells (BAECs). The upper panel shows EMMPRIN (red, left) and eNOS (green, center) and their merged image with DNA in blue (right). Arrows indicate where EMMPRIN and eNOS are located. The lower panel shows GOLPH3 (red, left), GM130 (green, center), and their merged image with nucleus in blue (right). Arrows indicate where GOLPH3 and GM130 are located. Scale bar; 10 µm. Images were taken using a Nikon E800 Microscope with a Plan-Fluorchromat 40×/0.75 objective (Nikon, Melville, NY). Shown are representative images from at least 3 independent experiments. (**B**) Immunolabeling of COS cells that were transfected with wild-type eNOS (blue, upper left panel), YFP-EMMPRIN (green, upper right panel) and HA-tagged GOLPH3 (red, lower left panel). The lower right panel shows their merged image. Scale bar; 10 µm. Arrows indicate eNOS-, EMMPRIN- and GOLPH3-rich areas. Scale bar; 10 µm. Shown are representative images from at least 3 independent transfection experiments. (**C**) Immunoprecipitation (IP) using anti-EMMPRIN (EMP) and total goat IgG from BAEC lysates. IP samples were blotted with eNOS and GOLPH3 antibodies. Input refers to eNOS levels in lysates before IP, indicating an equal amount of proteins in lysates used for IP. The blots are representative images from three independent experiments.

First, we determined the cellular localization of EMMPRIN and GOLPH3 in endothelial cells (Figure 3A). We found that EMMPRIN was co-localized with eNOS at the Golgi apparatus (Figure 3A, upper panel). Further, we observed that GOLPH3 was highly concentrated on the Golgi apparatus (Figure 3A, lower panel) as indicated by its co-localization with GM130, a Golgi marker. These results indicate that EMMPRIN and GOLPH3 co-localize with eNOS at the Golgi apparatus in endothelial cells. In addition to BAECs, we investigated their cellular localization in COS cells. We co-expressed eNOS with YFP-tagged EMMPRIN and HA-tagged GOLPH3 in COS cells. Consistent with our observation in endothelial cells, our results showed that these three proteins were co-localized on the Golgi membranes (Figure 3B).

We next examined if these proteins might be physically associated with each other in endothelial cells. Interestingly, EMMPRIN was co-immunoprecipitated with both eNOS and GOLPH3 in endothelial cells (Figure 3C, Figure S2). These results suggest that eNOS interacts with its substrates on the Golgi. EMMPRIN may act as a scaffold for eNOS and GOLPH3, forming a complex at the Golgi.

### EMMPRIN S-nitrosylation is increased in the aorta isolated from cirrhotic rats

**Figure 4:**
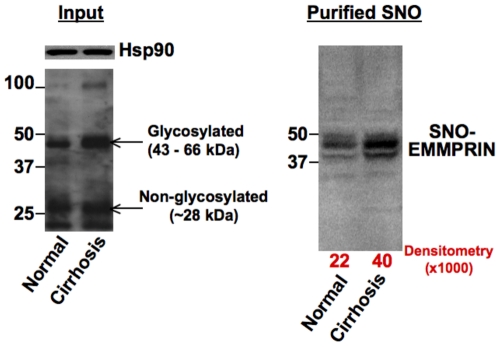
S-nitrosylation of EMMPRIN is increased in the aorta isolated from cirrhotic rats. Aorta samples were lysed in a lysis buffer. Three aorta samples were combined per group to obtain a sufficient amount of proteins for the biotin-switch assay. Lysates before the biotin-switch assay were blotted for EMMPRIN and a loading control, heat shock protein 90 (Hsp90) (input, left panel). Equal amounts of proteins (500 µg) in the lysates were used for the biotin-switch assay. Biotinylated proteins were captured by streptavidin agarose beads and blotted with an EMMPRIN antibody (right panel). The aorta from cirrhotic rats showed a higher level of S-nitrosylated EMMPRIN than that of normal rats. Interestingly, only those glycosylated EMMPRIN were S-nitrosylated in the aorta.

We also examined whether S-nitrosylation of EMMPRIN increases in pathological conditions where excessive eNOS-derived NO production is involved. We chose the aorta of cirrhotic rats with portal hypertension for this purpose, because in the arteries such as the aorta NO production is increased and extracellular matrix remodeling occurs [20], a potential role for EMMPRIN. Furthermore, the aorta provides a relatively large amount of proteins that are required for the biotin-switch assay to detect S-nitrosylation, compared to other arteries. EMMPRIN exists in glycosylated (an active form, 43–66 kDa) and non-glycosylated forms (∼28 kDa). The total EMMPRIN level in the aorta of cirrhotic rats was approximately 2-fold higher than that of the normal aorta (Figure 4, left panel). However, as indicated by Hsp90 content (loading control), the lysates prepared from the aorta of normal and cirrhotic rats had a similar protein content. Most interestingly, we found that S-nitrosylated EMMPRIN was also roughly 2-fold higher in the aorta of cirrhotic rats than that of normal rats and that glycosylated EMMPRIN was only S-nitrosylated (Figure 4, right panel).

## Discussion

Using a mass spectrometry-based approach, we identified for the first time nine Golgi resident and Golgi/ER-associated proteins from Golgi membranes isolated from rat livers that are potential targets for S-nitrosylation. The purity of the isolated Golgi membranes was reasonable. Although their enriched fractions showed a low level of ER contamination, it was not problematic since we identified Golgi/ER-associate proteins as well as Golgi proteins. Actually, ER contamination is almost inevitable since many ER resident proteins recycle through the Golgi where they are recognized by the KDEL receptor and retrieved back to the ER by COPI vesicles [21]. In fact, some researchers state that no organelle fractionation technique produces a “pure” preparation [22].

Importantly, among the nine Golgi resident and Golgi/ER proteins identified, we validated S-nitrosylation of EMMPRIN and GOLPH3 in endothelial cells by the biotin-switch assay coupled with Western blot. In addition, we found that EMMPRIN and GOLPH3 are basally S-nitrosylated and that S-nitrosylation of these proteins increases in response to eNOS stimulation. The immunofluorescence results show that EMMPRIN, GOLPH3 and eNOS are co-localized at the Golgi apparatus in endothelial cells. Furthermore, EMMPRIN was co-immunoprecipitated with both eNOS and GOLPH3, suggesting that these three proteins may form a complex at the Golgi apparatus. Collectively, these findings indicate that eNOS-derived NO modulates S-nitrosylation of EMMPRIN and GOLPH3 in endothelial cells.

eNOS is localized on the cytoplasmic side of the Golgi apparatus [4]–[6]. To our knowledge, the membrane topology of EMMPRIN at the Golgi apparatus is not known. However, given that glycosylation sites of EMMPRIN are located only on the N-terminus [16], [23], it is likely that the N-terminus is on the luminal side of the Golgi apparatus, while the C-terminus is on the cytoplasmic side. It is possible that eNOS-derived NO could S-nitrosylate the N-terminus of EMMPRIN by diffusing across the Golgi membrane. GOLPH3 is located on the cytoplasmic surface of the Golgi apparatus by binding to a membrane lipid, phosphatidylinositol 4-phosphate [PtdIns(4)P] [19], [24], [25]. It is possible that a complex of eNOS, EMMPRIN and GOLPH3 is formed on the cytoplasmic side of the Golgi apparatus through the C-terminus of EMMPRIN.

The mass spectrometry-based analysis also identified putative cysteine residues for S-nitrosylation on these Golgi resident and Golgi/ER-associated proteins. For example, cysteine 87 of EMMPRIN is an S-nitrosylated site. This residue is also known to form a disulfide bond and is located within the extracellular domain that is also known to induce matrix metalloproteinase 2 (MMP-2) expression/activation [16]–[18]. Thus, it is possible that S-nitrosylation of this cysteine residue may influence MMP-2 induction. The functional importance of Cys 288, a site for GOLPH3 S-nitrosylation identified in this study, has not been reported. Investigations on the effect of S-nitrosylation on protein function in relation to its cysteine residue will be highly appreciated.

EMMPRIN S-nitrosylation may have implication for pathological vasculature. It is known that the aorta and other arteries of cirrhotic rats exhibit excessive vasodilation due to elevated eNOS-derived NO production [26]–[28]. Further, increased NO level causes thinning of the aorta in cirrhotic rats, as an NOS inhibitor ameliorates aortic wall thickness [20]. These observations suggest that active vascular remodeling takes place in these arteries of cirrhotic animals and that increased eNOS-derived NO facilitates such arterial thinning in those animals. Since EMMPRIN is an inducer of MMPs and could be involved in vascular remodeling, we examined whether EMMPRIN is S-nitrosylated in the aorta isolated from cirrhotic rats. Interestingly, EMMPRIN S-nitrosylation was notably higher in the aorta of cirrhotic rats than that of normal rats. Importantly, only the glycosylated form of EMMPRIN is S-nitrosylated. Given that the Golgi is the major site of glycosylation and that eNOS is localized on the Golgi, this finding also supports that the primary site of EMMPRIN S-nitrosylation is the Golgi apparatus. Although the effect of S-nitrosylation on EMMPRIN functions is still not clear, it is possible that eNOS-derived NO enhances S-nitrosylation of mature EMMPRIN, induces MMP expression/activation and facilitates active arterial remodeling in cirrhotic animals.

Presently, the selectivity of S-nitrosylation of proteins in vivo is not completely understood. A recent study by Doulias et al. [29] has suggested that the selectivity of some proteins requires secondary structure elements and correct folding. As such glycosylation of EMMPRIN may be required for correct protein folding that will facilitate S-nitrosylation.

GOLPH3, also called GPP34, GMx33, MIDAS or yeast Vps74p, is known to be necessary for protein transport from the Golgi to the plasma membrane [19]. Using a temperature sensitive mutant protein, ts045-VSVG-EGFP, we previously demonstrated that eNOS localized to the Golgi apparatus delays protein transport from the Golgi apparatus to the plasma membrane, while a nuclear targeted form of eNOS does not affect the protein transport [7]. Thus, it is tempting to speculate that S-nitrosylation of GOLPH3 at the Golgi apparatus by eNOS-derived NO may play a role in the delayed protein trafficking observed in cells expressing wild-type eNOS.

The other seven Golgi resident and Golgi/ER-associated proteins that we identified are also known for their importance in cellular regulation. ERGIC-53 (also known as p58) and transmembrane emp24 domain-containing protein 9 (TMED9) are involved in protein trafficking between the ER and the Golgi apparatus [30], [31]. ERGIC-53 is an ER-Golgi intermediate compartment protein and serves as a molecular chaperone for the ER to the Golgi transport of a specific subset of secreted proteins such as coagulation factors V and VIII. Its defect causes a combined deficiency of coagulation factors V and VIII [31]. TMED9 is a member of p24 family [32]. It has been shown that p24 family members play an important role in retrograde transport from the Golgi to the ER by facilitating the formation of COPI-coated vesicles [30].

Giantin, a tail-anchored transmembrane protein with a large (350–380 kDa) N-terminal cytoplasmic region, is known to regulate Golgi structure. It is localized mostly at the rims of Golgi cisternae and influences diverse morphological changes of the Golgi by facilitating COPI vesicle formation and fusion [33], [34]. Further, four additional proteins, namely alpha-6-fucosyltransferase, fukutin related protein, polypeptide N-acetylgalactosaminyltransferase II and D-glucuronic acid C5-epimerase, are known to have implication in protein glycosylation.

As evidence is reported that NO impairs ERGIC-53 transport from the ER to the Golgi in macrophages [35], it is possible that these and other functions of these proteins are regulated by S-nitrosylation. Furthermore, besides the nine Golgi resident and Golgi/ER-associated proteins, we identified 69 potential S-nitrosylated proteins. They are considered cargo proteins and listed in Table S1. It is also possible that these cargo proteins are S-nitrosylated at the Golgi apparatus and distributed to their designated cellular location, which may subsequently impact the functions of these proteins. Given that the Golgi is an important site for post-translational modifications such as phosphorylation, glycosylation and sulfation, it may also be important for S-nitrosylation.

In conclusion, we have identified potential S-nitrosylated Golgi proteins, which are important regulators of extracellular matrix remodeling, signal transduction, protein trafficking, glycosylation and maintenance of the Golgi structure. Importantly, we have verified S-nitrosylation of EMMPRIN and GOLPH3 in endothelial cells, two example proteins among those identified. We have also showed a high prevalence of S-nitrosylated EMMPRIN in the aorta of cirrhotic rats. These findings provide new insights into organelle specific S-nitrosylation of proteins regulated by eNOS-derived NO.

## Supporting Information

Figure S1 **S-nitrosylation of Golgi membrane proteins is increased in a dose-dependent manner.** Tri-iodide chemiluminescence was used for the measurement of S-nitrosylated proteins formed in response to graded concentrations of NO donors, S-nitrosocysteine (Cys-NO) and diethylamine nitric oxide (DEANO).
(TIF)

Figure S2 **EMMPRIN forms a complex with eNOS and GOLPH3 in endothelial cells.** Bovine aortic endothelial cell (BAEC) lysates were immunoprecipitated using an anti-EMMPRIN (EMP) antibody or control (Goat) IgG followed by western blot for indicated proteins. This is a repeat of the experiment shown in Figure 3C but using a salt-containing wash buffer (50 mM Tris-HCl, pH7.5, 0.1 mM EDTA/EGTA, and.150 mM NaCl). EMMPRIN was co- immunoprecipitated with both eNOS and GOLPH3, although the efficiency was slightly reduced for eNOS by the addition of 150 mM salt in the wash buffer. This result confirms our finding in Figure 3C that EMMPRIN forms a complex with eNOS and GOLPH3 in endothelial cells.
(TIF)

Figure S3 **Inhibition of eNOS activity by N(G)-nitro-L-arginine methyl ester (L-NAME) decreases the formation of S-nitrosocysteines in endothelial cells.** Bovine aortic endothelial cells (BAECs) were incubated in cell culture media (Dulbecco's Modified Eagle's Media; DMEM) in the absence of fetal bovine serum (FBS) for 24 hours. Then 100 µM ATP was added to activate eNOS for 30 min (middle lane). Some cells were pretreated with 100 µM L-NAME in the absence of FBS for 24 hours before the addition of ATP (right lane). Cells were lysed, and 150 µg protein from each sample was analyzed by SDS-PAGE and western blotting using S-nitrosocysteine (rabbit, 1∶500, Sigma, St. Louis, MO, Cat#: N5411) and eNOS (mouse, 1∶1000, BD Biosciences, San Jose, CA, Cat#: 610296) antibodies. Proteins are S-nitrosylated at the base level without L-NAME and ATP treatment (left lane). The level of S-nitrosylated proteins is increased by ATP treatment (middle lane) but not when the cells are pretreated with L-NAME (right lane). Collectively, these results indicate that the increase in S-nitrosocysteine levels in BAECs is NOS activity dependent.
(TIF)

Table S1 **List of all the putative S-nitrosylated proteins from Golgi membrane samples.** We identified 78 putative S-nitrosylated proteins from our Golgi membrane samples, nine of which were Golgi resident and Golgi/ER-associated proteins (Table 1), while the rests were considered proteins in transit through the Golgi apparatus. This proteomic analysis also allowed us to identify the site where S-nitrosylation occurs on the protein.
(XLS)

## References

[1] Hess DT, Matsumoto A, Kim SO, Marshall HE, Stamler JS (2005). Protein S-nitrosylation: purview and parameters.. Nat Rev Mol Cell Biol.

[2] Stamler JS, Simon DI, Osborne JA, Mullins ME, Jaraki O (1992). S-nitrosylation of proteins with nitric oxide: synthesis and characterization of biologically active compounds.. Proc Natl Acad Sci U S A.

[3] Stamler JS (1994). Redox signaling: nitrosylation and related target interactions of nitric oxide.. Cell.

[4] Garcia-Cardena G, Martasek P, Masters BS, Skidd PM, Couet J (1997). Dissecting the interaction between nitric oxide synthase (NOS) and caveolin. Functional significance of the nos caveolin binding domain in vivo.. J Biol Chem.

[5] Garcia-Cardena G, Oh P, Liu J, Schnitzer JE, Sessa WC (1996). Targeting of nitric oxide synthase to endothelial cell caveolae via palmitoylation: implications for nitric oxide signaling.. Proc Natl Acad Sci U S A.

[6] Sessa WC, Garcia-Cardena G, Liu J, Keh A, Pollock JS (1995). The Golgi association of endothelial nitric oxide synthase is necessary for the efficient synthesis of nitric oxide.. J Biol Chem.

[7] Iwakiri Y, Satoh A, Chatterjee S, Toomre DK, Chalouni CM (2006). Nitric oxide synthase generates nitric oxide locally to regulate compartmentalized protein S-nitrosylation and protein trafficking.. Proc Natl Acad Sci U S A.

[8] Iwakiri Y (2011). S-nitrosylation of proteins: a new insight into endothelial cell function regulated by eNOS-derived NO.. Nitric Oxide.

[9] Greco TM, Hodara R, Parastatidis I, Heijnen HF, Dennehy MK (2006). Identification of S-nitrosylation motifs by site-specific mapping of the S-nitrosocysteine proteome in human vascular smooth muscle cells.. Proc Natl Acad Sci U S A.

[10] Wilson MC, Meredith D, Halestrap AP (2002). Fluorescence resonance energy transfer studies on the interaction between the lactate transporter MCT1 and CD147 provide information on the topology and stoichiometry of the complex in situ.. J Biol Chem.

[11] Wang Y, Taguchi T, Warren G, Celis J (2006). Purification of rat liver Golgi stacks..

[12] Jaffrey SR, Snyder SH (2001). The biotin switch method for the detection of S-nitrosylated proteins.. Sci STKE.

[13] Jaffrey SR, Erdjument-Bromage H, Ferris CD, Tempst P, Snyder SH (2001). Protein S-nitrosylation: a physiological signal for neuronal nitric oxide.. Nat Cell Biol.

[14] Bellinger AM, Reiken S, Carlson C, Mongillo M, Liu X (2009). Hypernitrosylated ryanodine receptor calcium release channels are leaky in dystrophic muscle.. Nat Med.

[15] Loureiro-Silva MR, Iwakiri Y, Abraldes JG, Haq O, Groszmann RJ (2006). Increased phosphodiesterase-5 expression is involved in the decreased vasodilator response to nitric oxide in cirrhotic rat livers.. J Hepatol.

[16] Biswas C, Zhang Y, DeCastro R, Guo H, Nakamura T (1995). The human tumor cell-derived collagenase stimulatory factor (renamed EMMPRIN) is a member of the immunoglobulin superfamily.. Cancer Res.

[17] Guo H, Zucker S, Gordon MK, Toole BP, Biswas C (1997). Stimulation of matrix metalloproteinase production by recombinant extracellular matrix metalloproteinase inducer from transfected Chinese hamster ovary cells.. J Biol Chem.

[18] Sun J, Hemler ME (2001). Regulation of MMP-1 and MMP-2 production through CD147/extracellular matrix metalloproteinase inducer interactions.. Cancer Res.

[19] Dippold HC, Ng MM, Farber-Katz SE, Lee SK, Kerr ML (2009). GOLPH3 bridges phosphatidylinositol-4- phosphate and actomyosin to stretch and shape the Golgi to promote budding.. Cell.

[20] Fernandez-Varo G, Ros J, Morales-Ruiz M, Cejudo-Martin P, Arroyo V (2003). Nitric oxide synthase 3-dependent vascular remodeling and circulatory dysfunction in cirrhosis.. Am J Pathol.

[21] Capitani M, Sallese M (2009). The KDEL receptor: new functions for an old protein.. FEBS Lett.

[22] Wu CC, MacCoss MJ, Mardones G, Finnigan C, Mogelsvang S (2004). Organellar proteomics reveals golgi arginine dimethylation.. Mol Biol Cell.

[23] Tang W, Chang SB, Hemler ME (2004). Links between CD147 function, glycosylation, and caveolin-1.. Mol Biol Cell.

[24] Kutateladze TG (2010). Translation of the phosphoinositide code by PI effectors.. Nat Chem Biol.

[25] Wood CS, Schmitz KR, Bessman NJ, Setty TG, Ferguson KM (2009). PtdIns4P recognition by Vps74/GOLPH3 links PtdIns 4-kinase signaling to retrograde Golgi trafficking.. J Cell Biol.

[26] Iwakiri Y, Cadelina G, Sessa WC, Groszmann RJ (2002). Mice with targeted deletion of eNOS develop hyperdynamic circulation associated with portal hypertension.. Am J Physiol Gastrointest Liver Physiol.

[27] Iwakiri Y, Groszmann RJ (2006). The hyperdynamic circulation of chronic liver diseases: from the patient to the molecule.. Hepatology.

[28] Tazi KA, Barriere E, Moreau R, Heller J, Sogni P (2002). Role of shear stress in aortic eNOS up-regulation in rats with biliary cirrhosis.. Gastroenterology.

[29] Doulias PT, Greene JL, Greco TM, Tenopoulou M, Seeholzer SH (2010). Structural profiling of endogenous S-nitrosocysteine residues reveals unique features that accommodate diverse mechanisms for protein S-nitrosylation.. Proc Natl Acad Sci U S A.

[30] Aguilera-Romero A, Kaminska J, Spang A, Riezman H, Muniz M (2008). The yeast p24 complex is required for the formation of COPI retrograde transport vesicles from the Golgi apparatus.. J Cell Biol.

[31] Nichols WC, Seligsohn U, Zivelin A, Terry VH, Hertel CE (1998). Mutations in the ER-Golgi intermediate compartment protein ERGIC-53 cause combined deficiency of coagulation factors V and VIII.. Cell.

[32] Strating JR, Martens GJ (2009). The p24 family and selective transport processes at the ER-Golgi interface.. Biol Cell.

[33] Sohda M, Misumi Y, Fujiwara T, Nishioka M, Ikehara Y (1994). Molecular cloning and sequence analysis of a human 372-kDA protein localized in the Golgi complex.. Biochem Biophys Res Commun.

[34] Toki C, Fujiwara T, Sohda M, Hong HS, Misumi Y (1997). Identification and characterization of rat 364-kDa Golgi-associated protein recognized by autoantibodies from a patient with rheumatoid arthritis.. Cell Struct Funct.

[35] Renna M, Faraonio R, Bonatti S, De Stefano D, Carnuccio R (2006). Nitric oxide-induced endoplasmic reticulum stress activates the expression of cargo receptor proteins and alters the glycoprotein transport to the Golgi complex.. Int J Biochem Cell Biol.

