# Supplementary figures and images for: Proteomic Identification of S-Nitrosylated Golgi Proteins: New Insights into Endothelial Cell Regulation by eNOS-Derived NO
Source: PLoS One. 2012 Feb 21;7(2):e31564. doi: 10.1371/journal.pone.0031564 (PMC3283662; doi:10.1371/journal.pone.0031564)

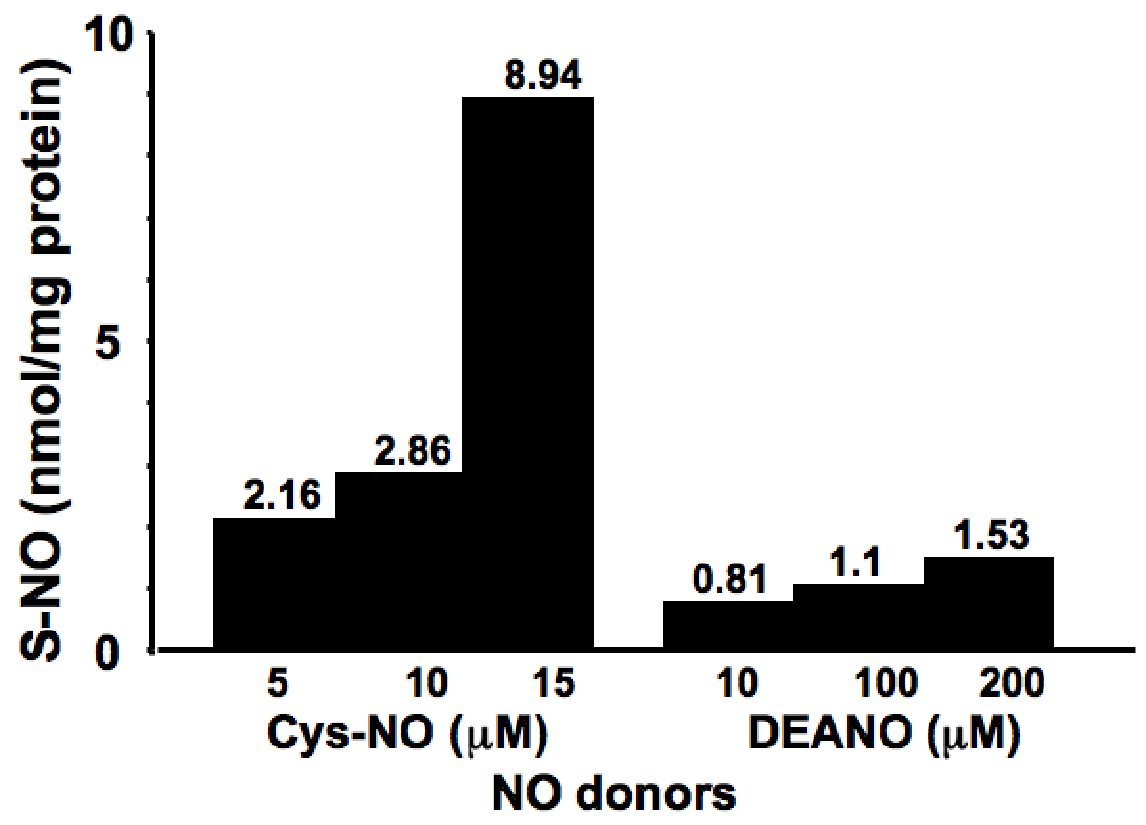

Supplement: Figure S1 — S-nitrosylation of Golgi membrane proteins is increased in a dose-dependent manner. Tri-iodide chemiluminescence was used for the measurement of S-nitrosylated proteins formed in response to graded concentrations of NO donors, S-nitrosocysteine (Cys-NO) and diethylamine nitric oxide (DEANO). (TIF) [file pone.0031564.s001.tif]

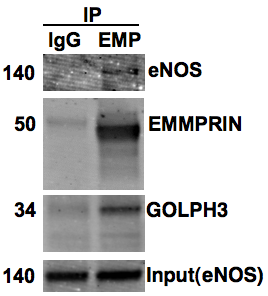

Supplement: Figure S2 — EMMPRIN forms a complex with eNOS and GOLPH3 in endothelial cells. Bovine aortic endothelial cell (BAEC) lysates were immunoprecipitated using an anti-EMMPRIN (EMP) antibody or control (Goat) IgG followed by western blot for indicated proteins. This is a repeat of the experiment shown in Figure 3C but using a salt-containing wash buffer (50 mM Tris-HCl, pH7.5, 0.1 mM EDTA/EGTA, and.150 mM NaCl). EMMPRIN was co- immunoprecipitated with both eNOS and GOLPH3, although the efficiency was slightly reduced for eNOS by the addition of 150 mM salt in the wash buffer. This result confirms our finding in Figure 3C that EMMPRIN forms a complex with eNOS and GOLPH3 in endothelial cells. (TIF) [file pone.0031564.s002.tif]

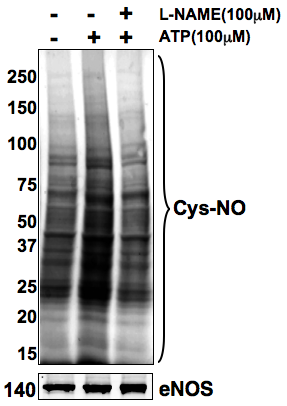

Supplement: Figure S3 — Inhibition of eNOS activity by N(G)-nitro-L-arginine methyl ester (L-NAME) decreases the formation of S-nitrosocysteines in endothelial cells. Bovine aortic endothelial cells (BAECs) were incubated in cell culture media (Dulbecco's Modified Eagle's Media; DMEM) in the absence of fetal bovine serum (FBS) for 24 hours. Then 100 µM ATP was added to activate eNOS for 30 min (middle lane). Some cells were pretreated with 100 µM L-NAME in the absence of FBS for 24 hours before the addition of ATP (right lane). Cells were lysed, and 150 µg protein from each sample was analyzed by SDS-PAGE and western blotting using S-nitrosocysteine (rabbit, 1∶500, Sigma, St. Louis, MO, Cat#: N5411) and eNOS (mouse, 1∶1000, BD Biosciences, San Jose, CA, Cat#: 610296) antibodies. Proteins are S-nitrosylated at the base level without L-NAME and ATP treatment (left lane). The level of S-nitrosylated proteins is increased by ATP treatment (middle lane) but not when the cells are pretreated with L-NAME (right lane). Collectively, these results indicate that the increase in S-nitrosocysteine levels in BAECs is NOS activity dependent. (TIF) [file pone.0031564.s003.tif]
